# An automatic diagnostic model for the detection and classification of cardiovascular diseases based on swarm intelligence technique

**DOI:** 10.1016/j.heliyon.2024.e25574

**Published:** 2024-02-05

**Authors:** C. Venkatesh, B.V. V. S. Prasad, Mudassir Khan, J. Chinna Babu, M. Venkata Dasu

**Affiliations:** a Department of Electronics and Communication Engineering, Annamacharya Institute of Technology and Sciences, Rajampet, AP, India; b School of Engineering (CSE), Anurag University, Hyderabad, India; c Department of Computer Science, College of Science & Arts, Tanumah, King Khalid University, P.O. Box: 960, Postal Code: 61421, Abha, Saudi Arabia

**Keywords:** Cardiovascular disease, Optimisation, Accuracy, Deep learning, Clinical data, Diagnosing

## Abstract

Globally, cardiovascular diseases (CVDs) rank among the leading causes of mortality. One out of every three deaths is attributed to cardiovascular disease, according to new World Heart Federation research. Cardiovascular disease can be caused by a number of factors, including stress, alcohol, smoking, a poor diet, inactivity, and other medical disorders like high blood pressure or diabetes. In contrast, for the vast majority of heart disorders, early diagnosis of associated ailments results in permanent recovery. Using newly developed data analysis technology, examining a patient's medical record could aid in the early detection of cardiovascular disease. Recent work has employed machine learning algorithms to predict cardiovascular illness on clinical datasets. However, because of their enormous dimension and class imbalance, clinical datasets present serious issues. An inventive model is offered in this work for addressing these problems.

An efficient decision support system, also known as an assistive system, is proposed in this paper for the diagnosis and classification of cardiovascular disorders. It makes use of an optimisation technique and a deep learning classifier. The efficacy of traditional techniques for predicting cardiovascular disease using medical data is anticipated to advance with the combination of the two methodologies. Deep learning systems can reduce mortality rates by predicting cardiovascular illness based on clinical data and the patient's severity level. For an adequate sample size of synthesized samples, the optimisation process chooses the right parameters to yield the best prediction from an enhanced classifier. The 99.58% accuracy was obtained by the proposed method. Also, PSNR, sensitivity, specificity, and other metrics were calculated in this work and compared with systems that are currently in use.

## 1 Introduction

Humans today live in a different economic and social context, and in order to address global dangers that could jeopardize the welfare state and freedom of movement, public and private strategies, decisions, and actions need to be redefined [1]. Changes in the average quality of the earth, water, space, and environment have negatively impacted human welfare and health in many communities across the world, causing societies to face a variety of serious health issues [2]. Cardiovascular disease (CVD) is another word for heart disease [3]. Cardiovascular disorders remain the most prevalent cause of death globally. As reported by the World Health Organisation (WHO), they contribute to 32% of all global fatalities, with an estimated 17.9 million deaths per year. More than 85 percent of all heart-related deaths are triggered by heart attacks, commonly known as myocardial infarction or MI [4]. A “cardiovascular disease” is a medical disorder which affects the walls of the coronary arteries, which get blocked with plaque and impede blood flow to the heart. It can result in chest pain or, in the worst-case scenario, a heart attack [5]. Cardiomyopathy, hypertension, inflammatory, coronary, valvular, ischemic and cardiovascular heart diseases are all examples of heart diseases [6]. There are numerous risk factors for heart disease that have been acknowledged or recognized and categorized into two groups. The variables which are immutable like patient's age, gender and family history are comes under the first group. On the other hand, patient's lifestyle falls on the second category [7]. There are multiple elements of cardiac illness that hinder the physiology or activity of the heart.

Doctors may struggle to quickly and effectively detect some cardiac disorders. As a result, it is vital to utilise computerised technology in cardiac disease detection to assist doctors in making faster and more accurate diagnoses [8]. The most significant component of clinical analysis of information is the forecasting of cardiac conditions from clinical data. In the world of information technology, innovations are occurring at a quick pace and are being introduced via multiple paths [9]. The positive impact of information technology on numerous facets of our lives today is enormous, and society cannot deny the significance of new technologies [10].

The amount of data available, particularly in health care, is enormous [11]. It can be challenging to recognise cardiovascular disease owing to indications like high cholesterol and blood pressure levels, irregular pulse rate, diabetes and additional ones. Cardiovascular disease (CVD) symptoms can differ across genders. A patient who is male endure chest pain, whereas a female patient could experience shortness of breath, Spartan tiredness and nausea in addition to chest pain. As a result, appropriate diagnosis and treatment have the potential to save countless lives [12]. Several large studies have been undertaken to find the predictive signs that indicate heart disease using participants' health data. Recent improvements in Deep Learning (DL) tools and approaches have boosted research in generating models and strategies for classifying heart disease [13].

Deep learning's breakthrough in speech recognition and computer vision is propelling its appeal [14,15]. Deep learning seeks to identify previously unknown links and patterns in data. The deployment of algorithms for data mining and machine learning in health care has ushered in the next phase of computing. Several data mining approaches have been widely utilised to diagnose cardiac disease [16]. The fundamental problem with machine learning systems is that effective feature engineering utilization, which can be a time-consuming effort. Deep learning has been extensively implemented to overcome the aforementioned difficulties in many assignments involving categorization in the health field, especially for cardiovascular disease [17,18]. The proposed method aims to minimize the financial burden of feature engineering while simultaneously providing an efficient strategy for boosting the dependability of classification results. The suggested study is centred on a cardiac dataset taken from a public data base. Before applying deep learning techniques, data is pre-processed. In terms of ECG signals, this can be accomplished utilising a variety of heart disease data sets. The novelty of the proposed approach makes use of robust pre-processing methods like buffering and fusion to ensure that there are no mistakes in classification and prediction. A large number of training sets are employed to increase prediction accuracy. Convolutional neural networks (CNN) and particle swarm optimisation (PSO) are utilised in this work to improve the detection and classification accuracy. One of the most successful classification systems for predicting cardiovascular events is the CNN.

## 2 Literature review

In the literature, researchers have proposed different machine learning-based diagnosis approaches for heart diseases. To emphasise the significance of the suggested work, the present study examines numerous available machine learning-driven diagnosis tools.

In 2023, Sivakannan Subramani et al. [19] proposed a machine learning-based cardiovascular disease prediction system. By merging the Heart Dataset with other classification models, these models take into consideration the data observation mechanisms and training procedures of a variety of different algorithms. The proposed approach has an accuracy of about 96%.

In 2023, Saadullah Farooq Abbasi et al. [20] developed a neonatal quiet sleep detection system based on CNN with decision support and EEG inputs. The authors used a CNN architecture that included pooling layers, two convolutional layers, and one ReLU layer in this model. A smoothing filter was also used to improve the long-term viability of the sleep stage classification. The proposed approach is 94.07% accurate.

In 2022, Saadullah Farooq Abbasi et al. [21] presented a Neonatal Sleep Stage Classification Using Ensemble Learning. In this model, the authors used an internet of things and developed ensemble-based automatic sleep stage classification, where Bagging and stacking ensembles are then used to integrate the results for final classification. The proposed approach has a 94.27% accuracy rate.

In 2022, Ghulab Nabi Ahmad et al. [22] developed a hybrid approach that utilised combination of machine learning techniques along with the Grid Search CV method. The as Per the analytical results, the extreme gradient boost Classifier with Grid Search CV has the greatest and nearly comparable testing and training accuracies of 94.21% and 95.03% respectively. Grid search has the issue of losing dimensionality when the number of hyper parameters assessed grows exponentially. However, there is no assurance that the search will result in the optimal option.

In 2022, Ghulab Nabi Ahmad et al. [23] proposed to forecast cardiac disease utilising a number of algorithms like KNN, SVM, and sequential feature selection method. The system relies on K-fold validation for verification. When employing cross-validation, train the model on as many training sets as possible. The significant computational cost of training on several training sets is one downside of utilising this strategy.

In 2022, Abdul Saboor et al. [24] devised a method for better forecasting human cardiac disease. The heart disease dataset was utilised to examine the method's performance using several performance metrics. To evaluate all the metrics nine machine learning classifiers are adopted before and after hyper parameter adjustment. This method resulted in significant gains in prediction classifier accuracy, which improved to 96.72%. Because the training method must be run k times from the beginning, the assessment procedure takes k times as long to complete.

In 2022, Victor Chang et al. [25] proposed an artificial intelligence model to detect the heart problems utilising machine. To diagnose cardiac issues more reliably, a random forest classifier approach is developed. The analysis of information is essential for this application, which is crucial because it outperforms training data by around 83%. The python libraries, which are a subset of the artificial intelligence model that use Python, are required for making predictions that SKLEARN is generally used in machine learning prediction. Random Forest delivers the finest values from the ML model, with the exception of the Decision Tree. It is the most basic strategy for accurately predicting heart disease. The disadvantage of the utilised classifier is that it consumes a lot of resources and processing power because it builds numerous trees to integrate their results. Training takes a long time since it combines many decision trees to determine the class.

In 2020, Sujatha et al. [26] established a framework for predicting cardiac illnesses that relied on KNN (K-nearest neighbours), decision trees, support vector machines, Nave Bayes, logistic regression and random forests approaches. The algorithms' performance was assessed using their precision, accuracy and F1-score values. Based on the experimental data, the Random Forest algorithm predicts heart disease more accuracy with a value of 83.52% than other classification algorithms, with an accuracy of 83.52%. Decision trees and forest algorithms have the disadvantage of being more unstable than other decision predictors and less effective at predicting the result of a continuous variable.

In 2020, Norma Latif Fitriyani et al. [27] proposed an efficient cardiovascular disease forecasting model based on DBSCAN (density-based spatial clustering of applications with noise). The suggested framework outperformed with accuracies of 93.90% and 94.40% for the StatLog and Cleveland datasets respectively. The disadvantages of using DBCSAN are that it cannot cluster large data sets well since the minPts-eps combination cannot be appropriately chosen for all clusters, and oversampling elevates the ADC output data rate significantly, causing setup and hold-time issues.

In 2020, Yuanyuan Pan et al. [28] developed the Enhanced Deep Learning-Assisted Convolutional Neural Network (EDCNN) to aid in enhancing patient predictions in cardiovascular diseases. This model relies on a more sophisticated multi-layer perceptron models with regularisation learning approaches that has been integrated into the Internet of Medical Things Platform (IoMT) for decision-support systems which enable clinicians to precisely diagnose heart patients' data on cloud platforms around the globe. This flexible architecture resulted in a precision of up to 94.1%. The drawbacks include their inability to encode object position and orientation, their failure to be spatially invariant to input data, and their high training complexity.

In 2020, Jian Ping Li et al. [29] claimed a machine learning-based strategy for diagnosing heart illness that is effective as well as precise. To remove unnecessary and redundant features, the authors used a variety of supervised classification methods as well as feature selection algorithms in their system. The experimental results indicate that the proposed feature selection approach (FCMIM) in conjunction with a classifier support vector machine can be used to create a high-level intelligent system for diagnosing heart disease. The proposed diagnosis system (FCMIM-SVM) achieved 92.37% accuracy. The drawbacks of artificial neural networks necessitate the use of processors with parallel processing power. The actualization of the equipment is thus dependent on this.

All of the methods discussed above do not use optimisation or deep learning techniques in their work. As a result, all of the approaches had an accuracy of less than 95%. To attain high accuracy, a hybrid model for the prediction and classification of cardiovascular illnesses relied on deep learning and optimisation techniques was developed in this work. The proposed research is motivated by the potential to enhance individual health outcomes, reduce healthcare expenditures, prevent problems, and ultimately beneficial influence the health of the public on a larger scale. Initial identification and treatment are critical to accomplishing the foregoing objectives.

The proposed approach initiates its operation by collecting ECG samples from various nodes and reshaping them into suitable sequence by properly buffering the ECG Pulses. The Buffered ECG pulses are fused appropriately by a multi-modal fusion framework to integrate all pulses of similar size and modalities. The fused ECG pulses are then sorted into distinct classes using K-Means depending on their pulse magnitudes. The classification characteristics are taken from the fused and clustered ECG pulses and submitted to the Convolutional Neural Network for anomalies detection and classification. For illness classification, the discovered abnormalities are again aggregated into variant groups. Finally, the classification results are shown in terms of psychovisual and quantitative metrics.

## 3 Proposed method

**Fig. 1:**
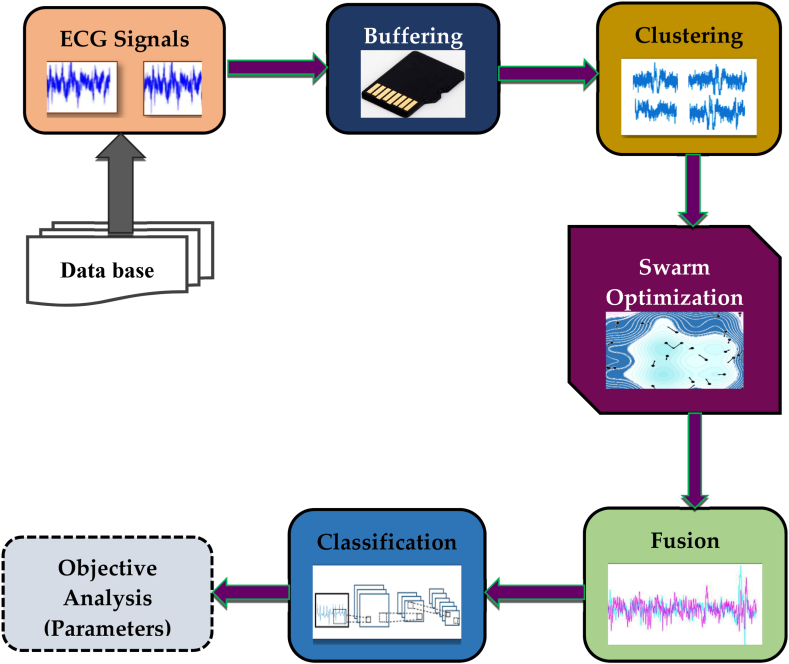
Block diagram of proposed method.

The proposed method which was created to categorise cardiovascular illnesses and to forecast heart disease with high accuracy is as shown in Fig. 1.

**Fig. 2:**
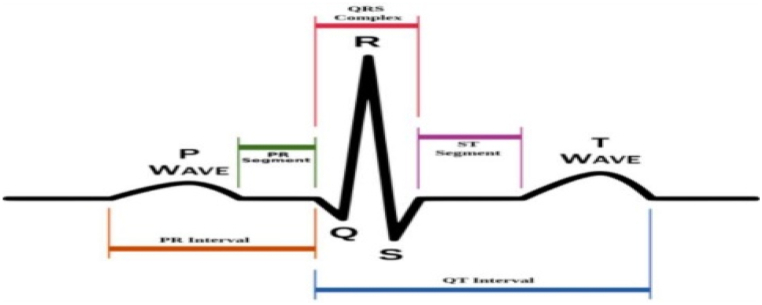
ECG signal.

The electrocardiogram (ECG), which analyses heart activity electro-physically in real time, is the most essential non-invasive physiological signal for CVD screening and diagnosis [30]. The electrocardiography (ECG) is an indicative instrument which tracks the electrocardiography (ECG) activity over the course of biological objects throughout a defined time interval. It acquires and preserves data from electrodes attached to the skin of defined biological events and records the required information in a precise way [31]. These variations are induced by waves known as T, S, R, Q and P waves (from right side) illustrated in Fig. 2. The 12-lead standard ECG has unipolar and bipolar limb leads.

The 12-lead ECG illustrates the heart's electrical activity in three dimensions as seen from 12 various vantage points [32]. When compared to 12-lead ECG data, single-lead ECG signal analysis involves less calculation and is easier to understand. However, the 12-lead ECG has following advantages: (1) It may identify indications of cardiac abnormalities in any heart location, assisting in the diagnosis of heart illness. (2) It may extensively portray the heart's core dynamics by providing detailed information about its behaviours. In this work, the ECG signals of ten patients from a private hospital and the signals of twenty patients from the MIT-BIH Arrhythmia database are collected. The signals are collected from the patient's different nodes, whereas a single ECG signal provides no further information.

### 3.1 B. buffering

Buffering is the technique of preloading data into a dedicated area of memory known as buffer memory. The buffer memory in the main memory acts as a temporary storage space for data transmitted between two or more devices, or between an application and a device [33]. To ensure proper sequencing, all signals are adjusted. ECG signal data is stored in databases. The ECG signals have been buffered and adjusted and are now ready for clustering.

### 3.2 C. Clustering

Data in a database can be grouped using a process known as clustering based on the qualities provided. Clustering does not need the grouping of a certain data class. Clustering can even be used to label the unidentified data class. Clustering is thus commonly classified as an unsupervised learning technique. One of the data grouping strategies employed in this paper is K-Means Clustering.

### 3.3 K-means clustering

The k-means clustering strategy suggested by Hartigan and Wong in 1979 [34] proved successful in resolving clustering challenges. In the aforementioned image segmentation, an unsupervised learning method is employed to differentiate the selected object from the background. The algorithm constructs ‘k' clusters from an image's input pixel set of *xy* size, where *x* and *y* reveal row and column respectively and $n_{\left(x,y\right)}$ reflects the input pixels to be clustered, with o denoting the cluster centre.

It estimates the distance by using eq (1) that is the shortest connecting each cluster.

(1) $$d=\|\left(x,y\right)-o_{j}∥$$

Where d is the distance across the centre of the cluster " o " and each pixel in an image. Furthermore, it allocates the centre to all pixels based on the distance " d ". The cluster centre is then calculated again using eq (2) until the halting criteria are met.

(2) $$f\left(j\right)=\sum_{j=1}^{c}\sum_{i=1}^{c}\left\|n_{\left(x,y\right)}-o_{j}\right\|$$

Where f(j) is the fitness function, and the number of clusters and cases ranges from 1 to “c". The letter ‘c' signifies the number of data elements. This function is dependent on another distance function computed with the ith case and the jth cluster.

### 3.4 D. Optimisation

The purpose of optimisation is to choose the best option from a limited set of options. When the possible options nurtures massively in proportion to the magnitude of the task, computation time becomes critical. A generalised optimisation problem is said to be solved when it can be addressed by maximising the value of the cost function [35]. Because the fusion method will not get a precise result, the optimisation technique is used to achieve the exact numbers. The particle swarm optimisation technique is employed to diagnose cardiovascular problems in this case.

#### 3.4.1 Cardiovascular disease detection using particle swarm optimisation

The PSO heuristic search approach was motivated by the swirling or collaborative actions exhibited by living groups. It is a strategy that uses intelligent evolutionary optimisation to iteratively improve a problem in order to improve a prospective solution in terms of a particular quality measure. Every particle, which consists of a fitness value derived by the optimisation function, speed, and position, is regarded as a possible solution to the optimisation problem. The employment of PSO thereby increases the speed of convergence [36]. This PSO approach is founded on five essential concepts like Adaptability, Proximity, Stability, Quality and Diverse Response.

A swarm of agents (particles) moves across the search space in pursuit of the optimal solution. Each particle has three parameters: speed, position, and the fitness value. Every atom or particle keeps track of its best response, best in class, or ${\;p}_{best}$,. The global best ($g_{best})$ is the best value of any particle. Every individual particle alters its orientation depending on its present position, speed, and distance from ${\;p}_{best}$, and current distance from $g_{best}$.

#### 3.4.2 Particle swarm optimisation algorithm

Unsupervised clustering and PSO, a swarm-based meta-heuristic approach, are combined to increase image segmentation quality. Let's start by making some assumptions about the variables. Set up a “population” of agents (particles) with equal distribution. Assess every particle's location in regard to the fitness function. when a particle's present position is superior compared to its earlier best position, it deserves to be updated. Determine the most optimal particle. Upgrade the particle velocities by using eq (3). Shift the particles to their new locations. Step 2 should be repeated until the halting criteria are met, as stated in eq (4).

(3) $$s_{i}^{t+1}=w.s_{i}^{t+1}+c_{1}u_{1}^{t}\left({pb}_{i}^{t}-l_{i}^{t}\right)+c_{2}u_{2}^{t}\left({gb}^{t}-l_{i}^{t}\right)$$

where, $s_{i}$:the particle's or agent's speed, w: weight of inertia, $c_{1}$:cognitive constant, $c_{2}$:social constant, $u_{1}$, $u_{2}$:random numbers, $p_{b}$:personal best and $g_{b}$:global best

(4) $$l_{i}^{t+1}=l_{i}^{t}+s_{i}^{t+1}$$

When $c_{1}=c_{2}=0$, the search space's border is reached, and each particle will sustain their current speed. As a result, in eq (5), S_ij_ is employed to calculate the velocity update. If $c_{1}$ > 0 and $c_{2}$ = 0, all particles are independent. Eq (6) shows the velocity update formula. When $c_{1}$ >0 and $c_{2}$ = 0, all particles in the swarm are drawn to a single point, and the update velocity changes to eq (7). When $c_{1}=c_{2}$, all particles are dragged to the average of $p_{best}$ and $g_{best}$.

(5) $$s_{ij}^{t+1}=s_{ij}^{t}$$

(6) $$s_{ij}^{t+1}=s_{ij}^{t}+{c_{1}r}_{1j}^{t}\left[p_{best,i}^{t}-x_{ij}^{t}\right]$$

where ${\;r}_{1}$, $r_{2}$: social constant, ${\;x}_{i}$:The particle's or agent's position

(7) $$s_{ij}^{t+1}=s_{ij}^{t}+{c_{2}r}_{2j}^{i}\left[g_{best}-x_{ij}^{t}\right]$$

**Fig. 3:**
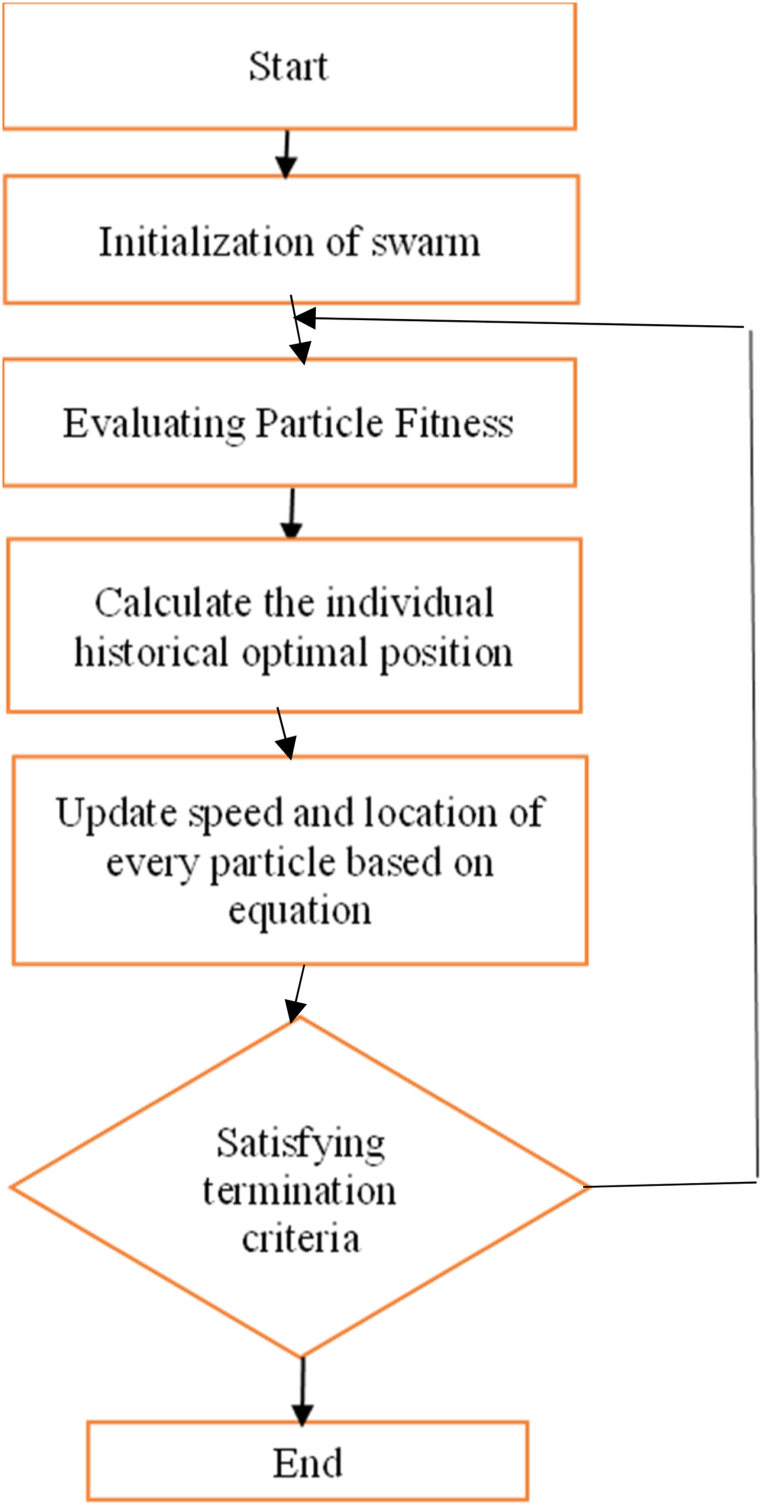
Flowchart of Particle swarm Optimisation.

The PSO method is primarily concerned with particle interactions. There are three distinct processing steps in this PSO technique. As illustrated in Fig. 3, they are the single particle travel route, the convergence, evolution and distribution issues of the entire particle system during a certain time period.

PSO The velocity and location of the ith particle is initialized as shown in eq (8)**.**

(8) $$x_{i}=\left(y_{i1},y_{i2}\;y_{iD}\right)$$

where $y_{iD}$ is the particle representing the Dth cluster centroid in solution. As a consequence, they exist high number of candidates available for swarm.

The fitness of the ith particle is determined as shown in eq (9).

(9) $$f\left(j\right)=\frac{\sum_{i=1}^{c}\sum_{j=1}^{c}\left\|n_{\left(x,y\right)}-o_{j}\right\|}{N_{x,y}}$$

Because it has been discovered that minimizing the fitness function can reduce cluster dispersion, minimizing the numeric value and number of data points is important.

Algorithm :

STEP1: Start the process
STEP2: Initialize the particles
STEP3: Verify the samples of particular parameters
STEP4: Calculate the individual's historical optimal position after evaluating
STEP5: Update the speed and location of every particle based on the equation.
STEP6Stop the process when the output meets the termination limits.
STEP7: End

### 3.5 E. Fusion

The act of combining information from multiple sources, including as sensor inputs, information processing units, databases, and knowledge bases, into a single representational structure is known as data fusion [37]. The fundamental purpose of data fusion in this work is to incorporate the favourable components of each single-modality method in order to improve it. Fusion can also be used to simulate or improve more complex results.

#### 3.5.1 Multimodal

The purpose of generating a fused picture is to combine functional and analytic data with varied perspectives of the heat source to aid in the localisation of the patient's symptoms and enhance the image's information quality. It has a multiplicative effect [38]. In this case, fusion is required to generate the final annotations list, which contains all heartbeats found using various physiological signals. Multimodal fusion combines ECG signals that have been buffered and changed based on magnitude and modalities.

### 3.6 F. Classification

By comparing the input signals to the training dataset, the disease should be classified as a heart attack, heart failure, heart valve, pericardial, or vascular disease. If the patient is in good health, it suggests that their heart is in good shape [39]. A new CNN model for predicting heart illness was built using ECG pictures. Following training, the newly suggested CNN model was used to extract characteristics from ECG images.

#### 3.6.1 Cardiovascular disease classification

Convolutional neural networks, a deep learning approach, were utilised in this study for predicting the probability of an individual obtaining cardiovascular disease (CVD). The suggested strategy concentrates on leveraging CNN to simulate temporal data for early-stage HF prediction [40]. Convolutional neural networks were utilised to differentiate between cardiovascular disease patients and healthy people [41]. Fundamentally, CNNs have multiple hyper parameters and specialised designs, which are costly and make selecting the optimum hyper parameter value difficult. Furthermore, CNNs are sensitive to the hyper parameter settings, could have a substantial impact on efficacy and behaviour of CNN designs [42].

**Fig. 4:**
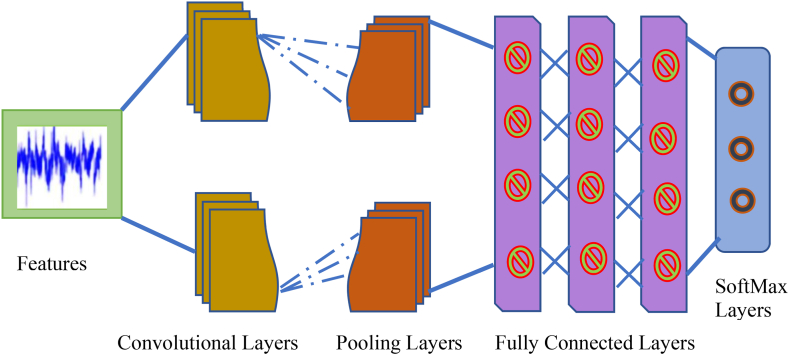
The architecture of CNN for prediction.

CNNs are neural networks with spectral layers that allow them to learn features at different levels. When compared to DNNs, three additional ideas—local filters, max-pooling, and weight sharing—produce a significant amount of power. The input layer only contributes to the shape of the input image, as seen in Fig. 4. CNN is made up of a few pairs of convolution and max-pooling layers that are used to generate the learnable parameters, as indicated in eq (10). A convolutional layer is always applied before a pooling layer. A max-pooling layer computes the maximum potential filter activation across a predefined window of sites. Convolutional neural networks are utilised at this step to generate lower-resolution features. The use of max-pooling has the benefits of decreasing positioning mistakes and hastening convergence. Because every neuron is coupled with every other neuron, the completely connected layer contains the most parameters compared to the other layers, as illustrated in eq (11). The classification inputs are then treated to the SoftMax activation function [43].

Number of parameters in a convolutional layer would be

(10) ((w*h*m)+1)

Where shape of width w, height h, previous layers filters m and filters k in the current layers.

Number of parameters in a fully connected layer would be

(11) ((c*p)+1*c)

Where c&p are neurons in present layer and earlier layer respectively.

The proposed CNN model diagnoses cardiovascular diseases substantially outstanding precision and is able to be employed as an extracting features tool for characteristic deep learning classifiers.

### 3.7 G. Attributes calculation

The statistics of sensitivity, specificity, and accuracy are widely used to define diagnostic tests. They are particularly useful for determining the quality and trustworthiness of a test [44].

#### 3.7.1 Accuracy

The accuracy of a diagnostic test affects how well it recognises and rules out a given condition. The accuracy of a diagnostic test is affected by sensitivity, specificity, and prevalence [45]. The accuracy was calculated by using eq (12).

(12) $$\text{Accuracy}=\frac{\left(\text{True}\;\text{Negatives}+\text{True}\;\text{Positives}\right)}{\left(\text{True}\;\text{Negatives}+\text{True}\;\text{Positives}+\text{False}\;\text{Negatives}+\text{False}\;\text{Positives}\right)}$$

#### 3.7.2 Sensitivity

The percentage of tests that result in true positives among all patients suffering from a disease is referred to as sensitivity. In other words, it is the ability of a test or an instrument to generate a favourable conclusion for a disease carrier. The ability to appropriately classify a test is critical. The sensitivity was calculated by using eq (13).

(13) $$\text{Sensitivity}=\frac{\left(\text{True}\;\text{Positives}\right)}{\left(\text{True}\;\text{Positives}+\text{False}\;\text{Negatives}\;\right)}$$

#### 3.7.3 Specificity

Specificity is defined as the ability to clearly evaluate and analyse in the presence of components that could be expected to be present. Degradants, matrix, and contaminants are usually included. The formula for calculating specificity is shown in eq (14).

(14) $$\text{Specificity}=\frac{\left(\text{True}\;\text{Negatives}\right)}{\left(\text{Flase}\;\text{Positives}+\text{True}\;\text{Negatives}\;\right)}$$

#### 3.7.4 Positive predictive value

If a person receives a positive test result, the positive predictive value estimates their likelihood of having the disease, condition, biomarker, or mutation (change) in the gene being examined. The positive predictive value of a test can be determined using eq (15).

(15) $$\text{PPV}=\frac{\left(\text{True}\;\text{Positives}\right)}{\left(\text{True}\;\text{Positives}+\text{False}\;\text{Positives}\;\right)}$$

#### 3.7.5 Negative predictive value

The likelihood that a person does not have the disease, condition, test-related biomarker, or gene mutation (change) that resulted in a negative result is determined by negative predictive value. The negative predictive value of a test can be used to measure its accuracy by using eq (16).

(16) $$\text{NPV}=\frac{\left(\text{True}\;\text{Negatives}\right)}{\left(\text{Flase}\;\text{Negatives}+\text{True}\;\text{Negatives}\;\right)}$$

## 4 Simulation resluts

In this study, an efficient deep learning-based categorization system for cardiovascular disorders such as heart attack, heart failure, heart valve, and vascular diseases was constructed. To begin, ECG signals are collected in order to diagnose and classify cardiovascular disorders. This study deployed ECG signals from the MIT-BIH Arrhythmia database.

**Fig. 5:**
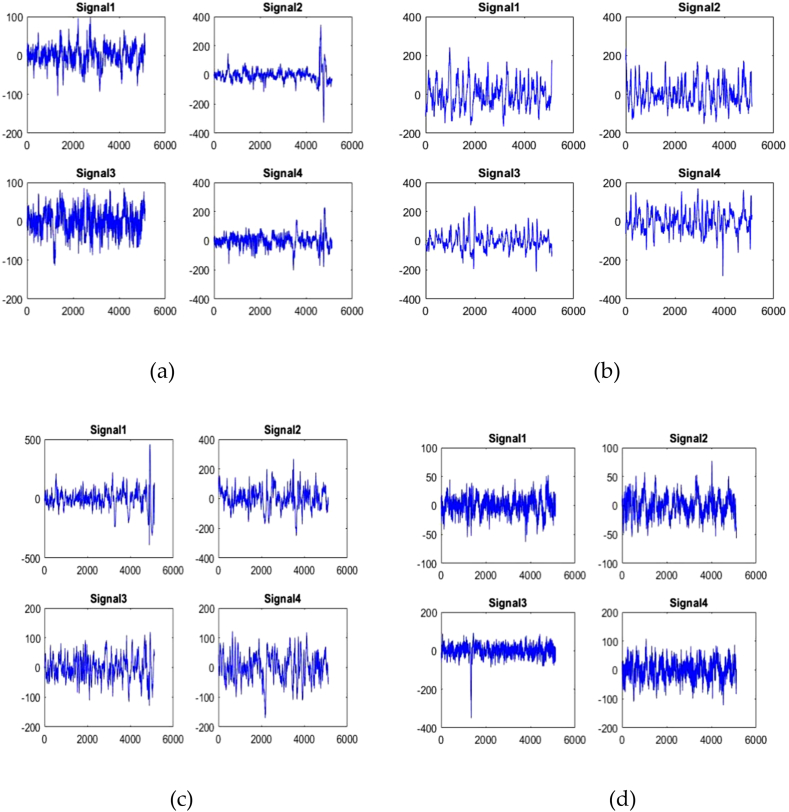
ECG signals of (a) Inpatient-1 (b) Inpatient-2 (c) Inpatient-3 (d) Inpatient-4.

An ECG is a non-invasive diagnostic procedure that captures the heart's electrical activity over time. ECG readings offer important insights into the heart's rhythm and can aid in the diagnosis of a number of cardiac conditions. This study gathered ECG signals from 12 patients from various nodes in the MIT-BIH Arrhythmia database. Fig. 5 illustrates ECG signals taken from various nodes of four inpatients 1, 2, 3 and 4.

**Fig. 6:**
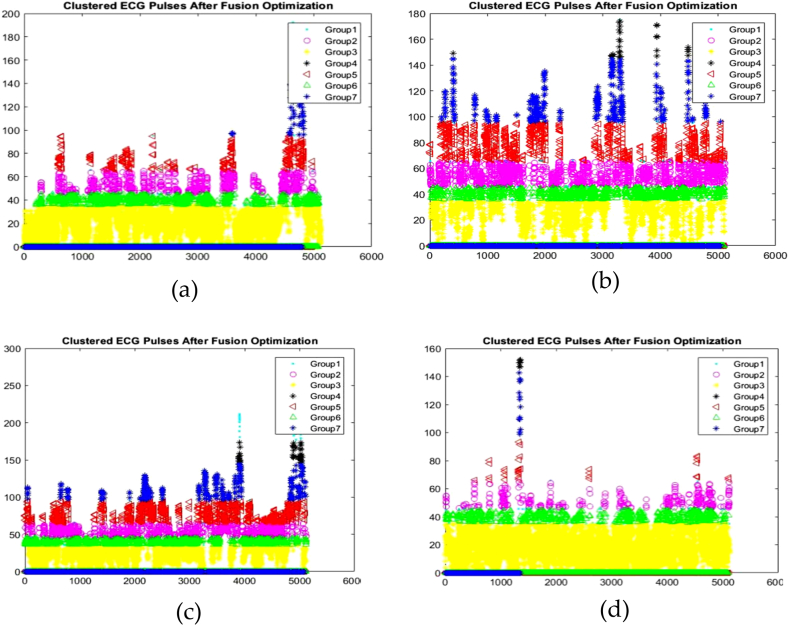
Clustered ECG signals of (a) Inpatient-1 (b) Inpatient-2 (c) Inpatient-3 (d) Inpatient-4.

The recorded ECG signals must be temporarily stored before being utilised in a subsequent step. Buffering is the process of temporarily storing data in a memory region. The buffered data must be grouped, which can be accomplished throughout the clustering phase. Clustering divides a population or set of data points into groups so that the data points within each group are more similar to one another and dissimilar from the data points in the other groups. To obtain the desired value, K Means clustering is utilised. Using k means approaches, data points in a dataset are clustered based on their nearest mean values. The k means clustering algorithm can be used to determine the best arrangement of data points into clusters while keeping the distance between points in each cluster to a minimum. Fig. 6 depicts the ECG signals of four inpatients 1, 2, 3 and 4 that have been clustered based on the nearest value.

**Fig. 7:**
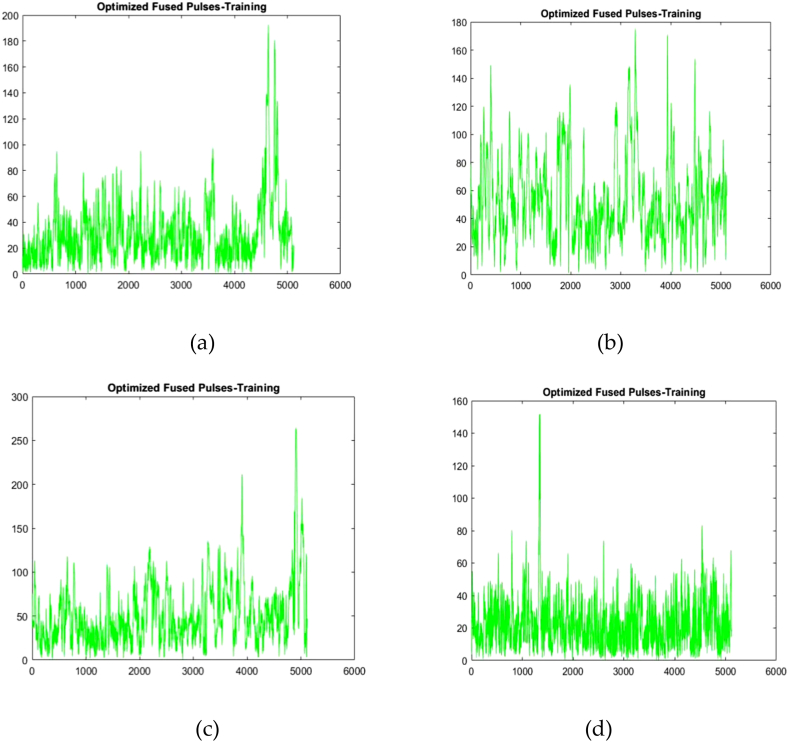
Optimised fused pulses of (a) Inpatient-1 (b) Inpatient-2 (c) Inpatient-3 (d) Inpatient-4.

Because the clustered pulses do not offer the correct value, the optimisation technique is applied to acquire an exact value. The purpose of optimisation is to increase the accuracy of the model while decreasing the likelihood of errors. The particle swarm optimisation (PSO) technique is employed in this work to identify the optimal solution, which has a high-dimensional search space and particle interaction. The clustered data of two patients is then subjected to an optimisation process to obtain the desired value. Fig. 7 depicts the optimised pulses of four inpatients 1, 2, 3 and 4.

**Fig. 8:**
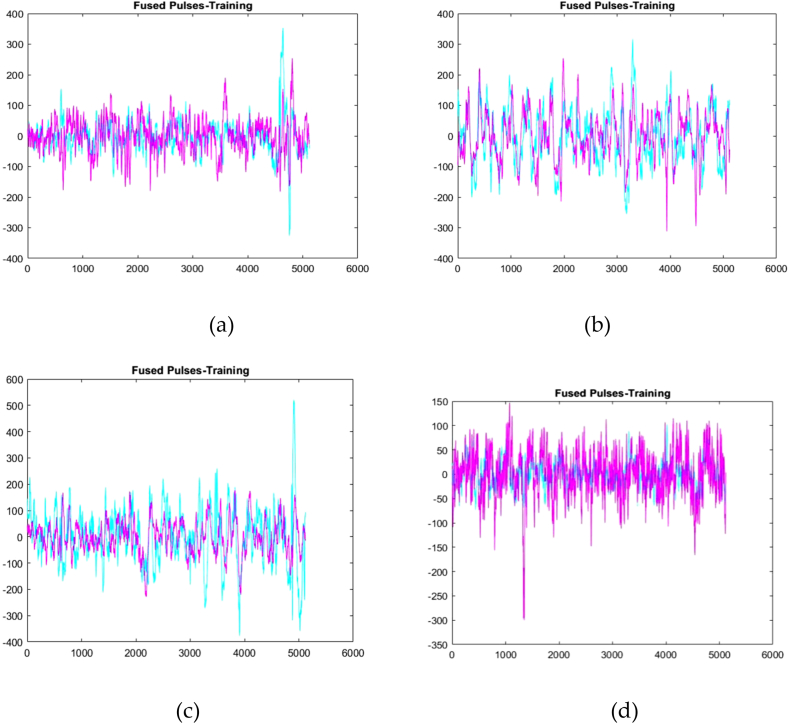
Fused pulses of (a) Inpatient-1 (b) Inpatient-2 (c) Inpatient-3 (d) Inpatient-4.

The ECG signals are fused once they have been optimised. Data fusion is the process of merging data from several modalities in order to obtain complementary and more comprehensive information for deep learning models that outperform those that solely use one data modality. Multimodal fusion is used to merge data from many sheathes into a single command. Fig. 8 depicts the fused pulses of inpatients 1, 2, 3 and 4.

**Fig. 9:**
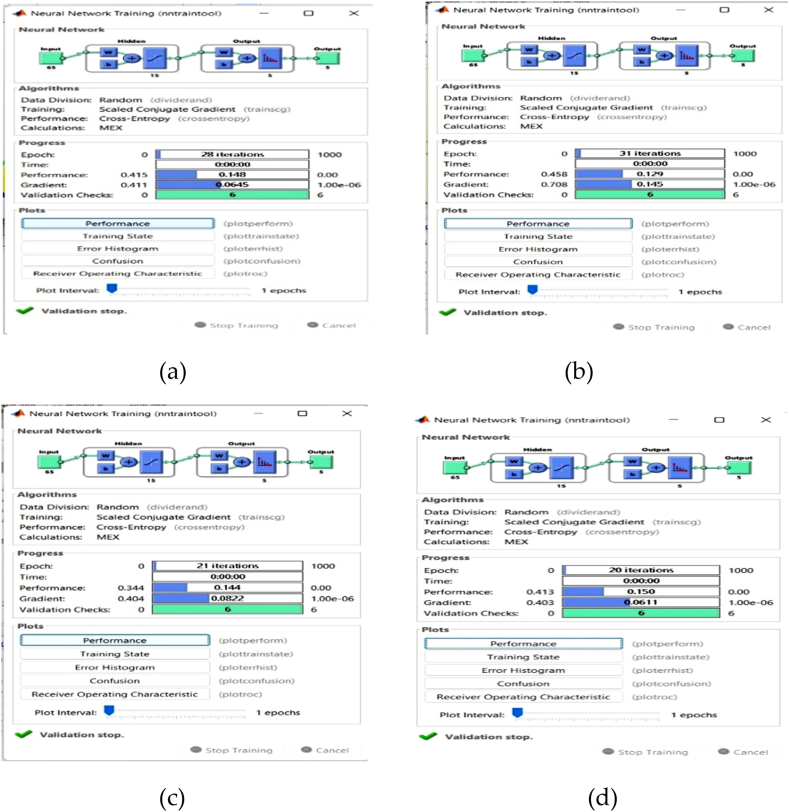
Training phases of (a) Inpatient-1 (b) Inpatient-2 (c) Inpatient-3 (d) Inpatient-4.

**Fig. 10:**
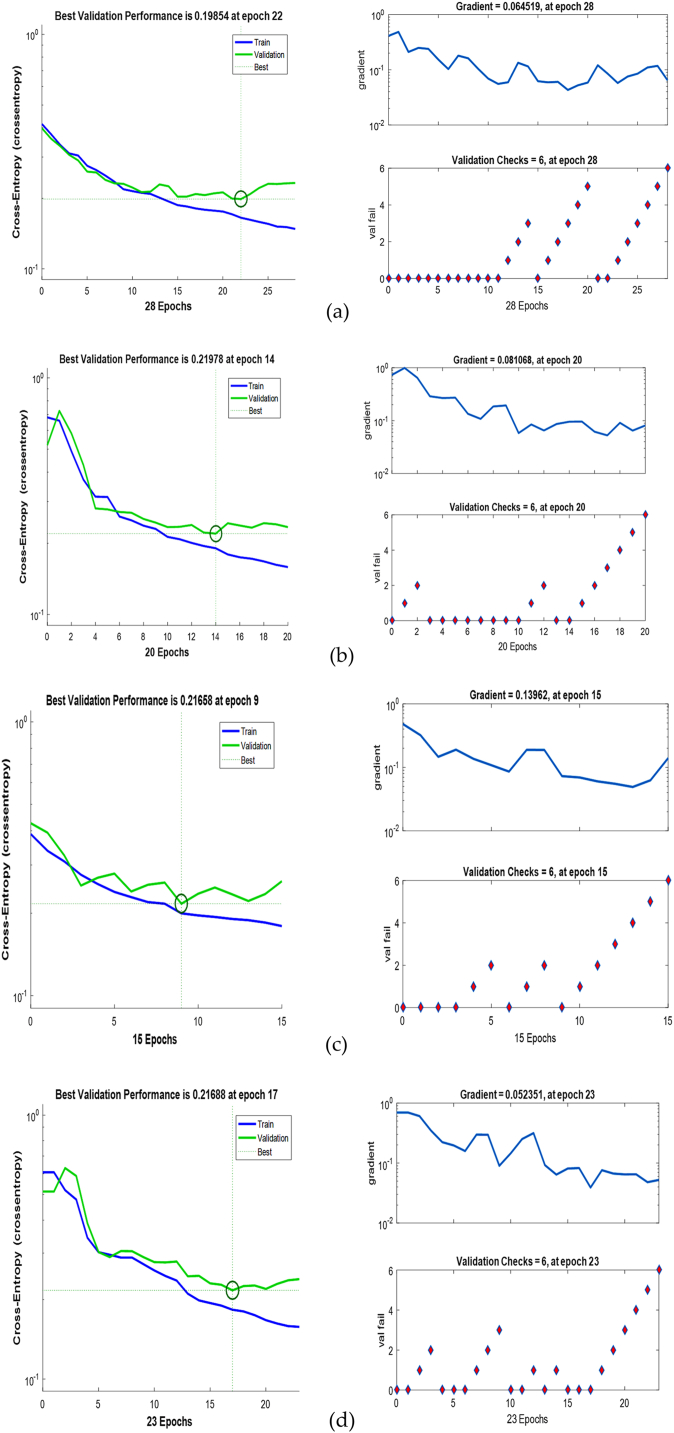
Validation performance and training status of (a) Inpatient-1 (b) Inpatient-2 (c) Inpatient-3 (d) Inpatient-4.

After all of the features have been extracted, they are fed into the classification process, where CNN is used to classify cardiovascular diseases. It can learn, save, and make connections between inputs and outputs. The neural network begins its operation by adjusting the weights associated with all correlations. Certain data sets were used to train the network. Fig. 9 depicts the neural network training phase. Fig. 10 depicts the total number of intervals utilised to train the features acquired from clusters for four inpatients 1, 2, 3 and 4.

**Fig. 11:**
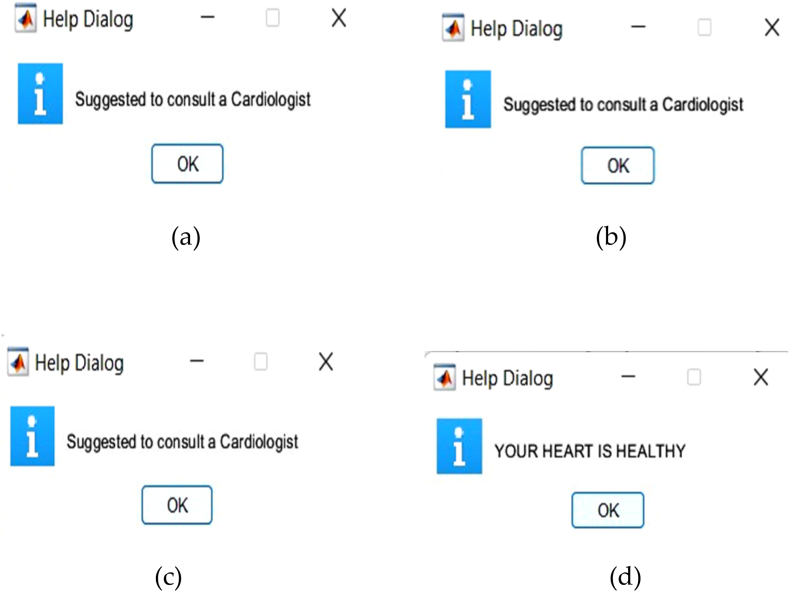
Help dialogue boxes of (a) Inpatient-1 (b) Inpatient-2 (c) Inpatient-3 (d) Inpatient-4.

After classification of the diseases, the results are validated by constructing the features as cross entropy and gradient. In the validation performance curve and training state if the cross entropy and gradient values are approximately zero then the model is perfectly trained otherwise not trained well. Best line should be a dotted line, best line represents that other line should lie on or near these lines then we can confirm that training has to be done successfully. Any line meets or passes near to the best dotted line it means the convergence has achieved. Cross Entropy feature is used in classification tasks. The cross entropy and gradient values of inpatient-1, inpatient-2, inpatient-3 and inpatient-4 are shown in Fig. 10. CNN not only categorises the disease, but also provides information such as “suggested to consult a doctor” if any abnormalities are present else it provides information as “Your Heart is Healthy”. Fig. 11 depict the corresponding dialogue boxes for inpatient-1, inpatient-2, inpatient-3 to consult a doctor and inpatient-4 heart is healthy.

**Fig. 12:**
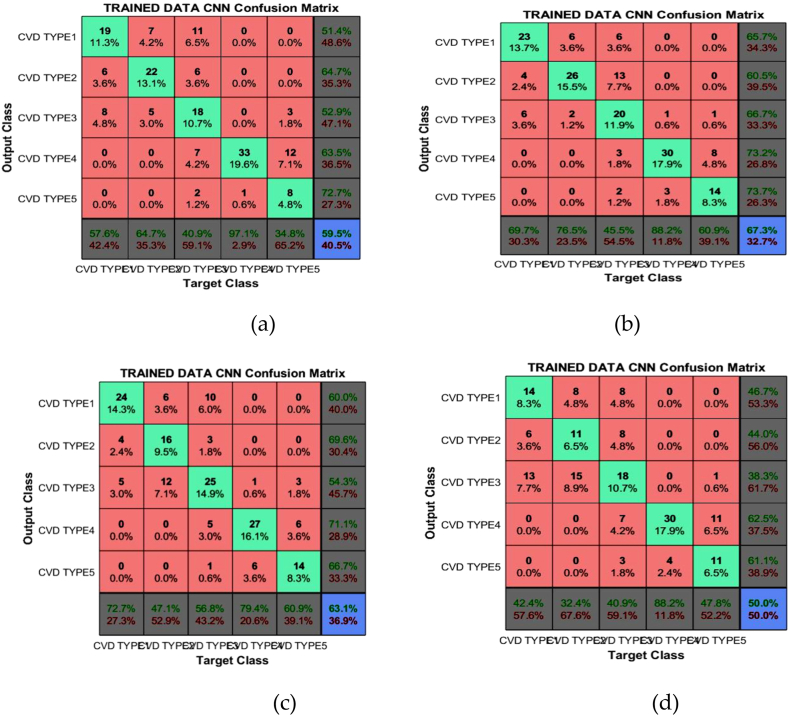
Confusion matrix of (a) Inpatient-1 (b) Inpatient-2 (c) Inpatient-3 (d) Inpatient-4.

A classification algorithm's performance is determined by the confusion matrix. It reveals how many correct and incorrect predictions the model generated. The diagonal matrix depicts the distribution of correct prophecies for each class. Fig. 12 depicts the confusion matrix for inpatients 1, 2, 3 and 4.

**Fig. 13:**
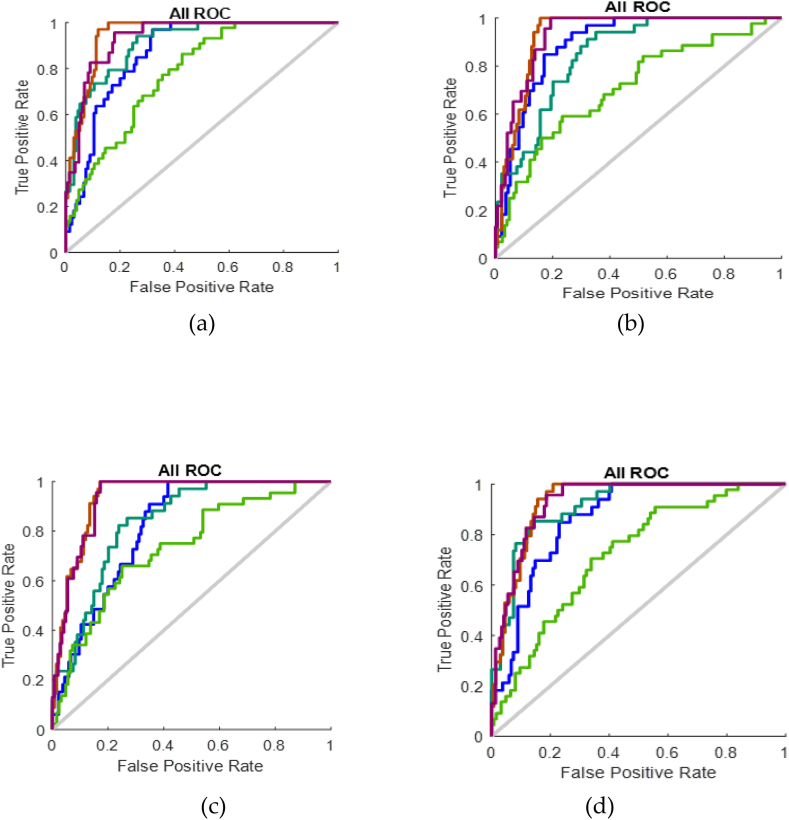
Receiver operating characteristics of (a) Inpatient-1 (b) Inpatient-2 (c) Inpatient-3 (d) Inpatient-4.

Lastly, the ROC curve has been used to assess the efficacy and reliability of the model that was created. Fig. 13 depicts the operative ROC curves for all four inpatients 1, 2, 3 and 4.

The trade-off involving the percentage of true positives and the false positive rate can be observed by the receiver operating characteristics. All the receiver operating characteristics are obtained after training, validation and testing. If the ROC curve was close to one, that means the system was perfect.

**Fig. 14:**
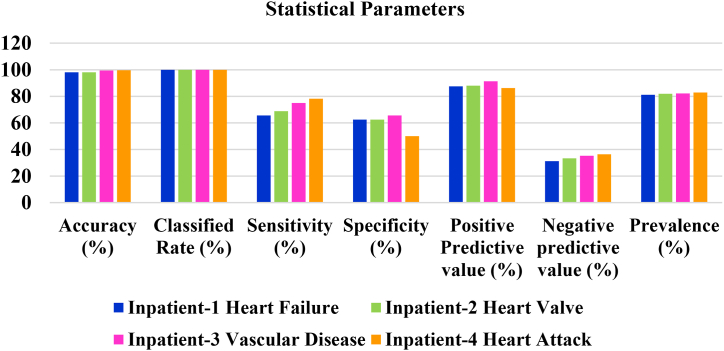
Graphical Representation of statistical Parameters.

In Table 1, it clearly observed that the proposed model classifies the inpatient-1disease as heart attack and inpatient-2 disease as vascular disease from their corresponding ECG signals. The current study's major goal was to improve the accuracy of categorization models. This method has demonstrated to be a highly dependable source for heart disease predictions, with 98.03%, 98.100%, 99.420% and 99.580% accuracy for inpatient-1, inpatient-2, inpatient-3 and inpatient-4 respectively. The graphical representation of statistical parameters for all the four inpatients is shown in Fig. 14.

**Table:1:** Statistical Parameters of inpatient-1, inpatient-2, inpatient-3 and inPatient-4.

Parameter	**Inpatient-1**	**Inpatient-2**	**Inpatient-3**	**Inpatient-4**
Detected Heart Disease (Type)	Heart Failure	Heart Valve	Vascular Disease	Heart Attack
Accuracy (%)	98.030	98.100	99.420	99.5800
Classified Rate (%)	100.000	100.000	100.000	100.000
Sensitivity (%)	65.625	68.750	75.000	78.125
Specificity (%)	62.500	62.500	65.625	50.000
Positive Predictive value (%)	87.500	88.000	91.304	86.206
Negative predictive value (%)	31.250	33.333	35.294	36.363
Prevalence (%)	81.200	81.870	82.120	82.860

**Fig. 15:**
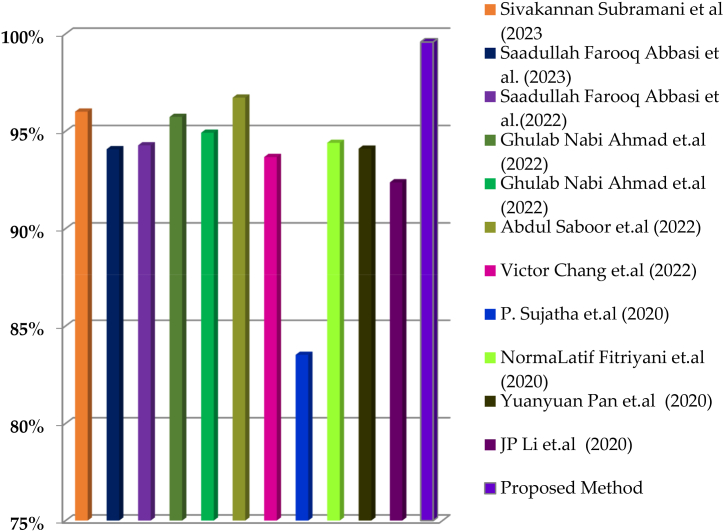
Graphical Representation of comparative results.

In Table 2, it is clearly observed that the proposed method classifies the cardiovascular diseases with an accuracy of 99.58% which is higher than the conventional methods which are mentioned in the literature review. Also, the proposed method proved to be the best by comparing the other parameters with the existing methods. The graphical representation of the comparative results is shown in Fig. 15. From the figure it is clearly observed that the proposed method yields high accuracy compared to the existing methods.

**Table:2:** Comparative results.

S.No	YEAR	AUTHORS	METHOD	ACCURACY
1.	2023	Sivakannan Subramani et al.	Machine learning Models	96%
2.	2023	Saadullah Farooq Abbasi et al.	CNN with decision support system	94.07%
3.	2022	Saadullah Farooq Abbasi et al.	CNN with Ensemble Learning	94.27%
4.	2022	Ghulab Nabi Ahmad et al.	KNN with Gridsearchcv	95.73%
5.	2022	Ghulab Nabi Ahmad et al.	KNN with Sequential Feature Selection	94.92%
6.	2022	Abdul Saboor et al.	SVM	96.72%
7.	2022	Victor Chang et al.	Artificial intelligence model	93.67%
8.	2020	P. Sujatha et al.	Supervised Machine Learning Algorithms	83.52%
9.	2020	NormaLatif Fitriyani et al.	Clinical Decision Support System	94.40%
10.	2020	Yuanyuan Pan et al.	EDLACNN	94.1%
11.	2020	JP Li et al.	FCMIM-SVM	92.37%
12.	**Proposed Method**			**99.58%**

## 5 Conclusion

Cardiovascular disease (CVD) is the most prevalent cause mortality in both men and women throughout the world. The rapid spread of these diseases and their sequelae has a negative impact on societies and places a substantial financial and physical strain on the worldwide community. For the diagnosis and classification of cardiovascular ailments, a hybrid model based on deep learning techniques such as CNN and particle swarm optimisation was developed. The proposed system achieves 99.58% detection and 100% classification accuracy. For future work, the proposed method is implemented to identify more cardiovascular diseases and also work on large datasets to improve accuracy. Also, other deep learning approaches algorithms can be introduced into Internet of Things (IoT) environments in the future, allowing for more accuracy in terms of results and potentially saving several human lives.

**Policy Recommendation:** Based on these findings, we advise doctors to perform periodic inspections in order to get the most out of the interventions. It's also advised to support local public health offices and radiologists.

Implications: The findings of our research imply that procedures should stress the significance of affording families emotional assistance. Interpreting ECG signals entails skills, and clinicians examine a variety of factors, such as the shape and duration of waves, intervals, and segments.

## Funding

This research is not funded.

## Data availability statement

Data will be made available on request.

## CRediT authorship contribution statement

**C. Venkatesh:** Writing – original draft, Formal analysis, Data curation, Conceptualization. **B.V. V. S. Prasad:** Writing – original draft, Visualization, Validation, Data curation. **Mudassir Khan:** Writing – original draft, Supervision, Software, Resources, Project administration, Conceptualization. **J. Chinna Babu:** Validation, Software, Project administration, Methodology, Investigation. **M. Venkata Dasu:** Writing – original draft, Visualization, Validation, Project administration, Methodology.

## Declaration of competing interest

The authors declare the following financial interests/personal relationships which may be considered as potential competing interests:Mudassir Khan reports a relationship with 10.13039/501100007446King Khalid University that includes: non-financial support. The authors declare no conflict of interest. If there are other authors, they declare that they have no known competing financial interests or personal relationships that could have appeared to influence the work reported in this paper.
